# *Bacillus velezensis* BV01 Has Broad-Spectrum Biocontrol Potential and the Ability to Promote Plant Growth

**DOI:** 10.3390/microorganisms11112627

**Published:** 2023-10-25

**Authors:** Ting Huang, Yi Zhang, Zhihe Yu, Wenying Zhuang, Zhaoqing Zeng

**Affiliations:** 1 State Key Laboratory of Mycology, Institute of Microbiology, Chinese Academy of Sciences, Beijing 100101, China; h_ting17@163.com (T.H.); zhangyicrazy@163.com (Y.Z.); zhuangwy@im.ac.cn (W.Z.); 2 College of Life Sciences, Yangtze University, Jingzhou 434025, China; zhiheyu@hotmail.com

**Keywords:** *Bacillus*, antifungal activity, fungal phytopathogens, wheat root rot, *Fusarium* wilt, greenhouse pot experiment

## Abstract

To evaluate the potential of a bacterial strain as a fungal disease control agent and plant growth promoter, its inhibitory effects on phytopathogens such as *Bipolaris sorokiniana*, *Botrytis cinerea*, *Colletotrichum capsici*, *Fusarium graminearum*, *F*. *oxysporum*, *Neocosmospora rubicola*, *Rhizoctonia solani*, and *Verticillium dahliae* were investigated. The results showed that the inhibitory rates in dual-culture and sterile filtrate assays against these eight phytopathogens ranged from 57% to 83% and from 36% to 92%. The strain was identified as *Bacillus velezensis* based on morphological and physiological characterization as well as phylogenetic analyses of *16S* rRNA and the gyrase subunit A protein (*gyrA*) regions. The results demonstrated that *B. velezensis* was able to produce fungal cell-wall-degrading enzymes, namely, protease, cellulase, and β-1,3-glucanase, and the growth-promotion substances indole-3-acetic acid (IAA) and siderophore. Furthermore, *B*. *velezensis* BV01 had significant control effects on wheat root rot and pepper *Fusarium* wilt in a greenhouse. Potted growth-promotion experiments displayed that BV01 significantly increased the height, stem diameter, and aboveground fresh and dry weights of wheat and pepper. The results imply that *B*. *velezensis* BV01, a broad-spectrum biocontrol bacterium, is worth further investigation regarding its practical applications in agriculture.

## 1. Introduction

Crop diseases caused by phytopathogens have resulted in a decrease in agricultural yields and quality, leading to significant economic losses [1]. In particular, soil-borne fungal infections of important crops such as wheat, corn, rice, and pepper cause large economic losses [2]. The United Nations 2030 Sustainable Development Goals suggested that the world should ensure sustainable consumption and production patterns, promote sustainable agriculture, and reduce environmental pollution [3]. For a long time, synthetic chemical pesticides were commonly used in traditional agriculture to combat plant diseases, but they often caused environmental pollution and residual toxic effects in animals and humans [4]. Thus, discovery of eco-friendly, long-lasting, and effective methods are required for disease prevention and management in agriculture. The use of microbial and biochemical agents has been explored as a practical alternative approach [5].

The plant-growth-promoting rhizobacteria (PGPRs) are often used for the production of bioactive substances that can protect plants by suppressing pathogens, inducing systemic resistance, or improving resistance to environmental stresses, by facilitating nutrient acquisition and modulating phytohormone levels in plants [6,7]. In recent years, *Bacillus subtilis* and its closest relatives *B*. *amyloliquefaciens*, *B*. *velezensis*, *B*. *cereus*, and *B*. *licheniformis* have been widely used as biofertilizers and biofungicides [8,9]. *Bacillus velezensis* FZB42, the classical PGPR strain, was successfully used as a biocontrol agent in potato, strawberry, wheat, and cabbage [10,11,12,13,14]. The most prevalent plant fungal diseases, such as grey mold, *Fusarium* head blight, anthracnose, and root rot, etc., are mainly caused by species of *Botrytis*, *Fusarium*, *Colletotrichum*, and *Rhizoctonia* [15]. This can be attributed to their broad host range, genetic diversity, rapid adaptation to plant disease resistance, and production of toxins [16]. Previous studies have shown that *B*. *velezensis* is a promising agent for control of *Rhizoctonia solani* [17], *Gaeumannomyces graminis* var. *tritici* [18], *Fusarium oxysporum* f. sp. *niveum* [19], *Botrytis cinerea*, *Colletotrichum gloeosporioides*, and *Phytophthora infestans* [20], and it has attracted widespread attention in agricultural disease research. Nevertheless, studies on its biocontrol mechanism, screening of excellent strains, analyses of transcriptomics, proteomics, metabolomics, and research on industrial and commercial applications of *B*. *velezensis* are needed [21].

In this study, we aimed to assess the potential of a newly isolated bacterial strain, *B*. *velezensis* BV01, as a broad-spectrum biocontrol agent and investigate its capacity to control plant diseases and promote wheat and pepper development. The findings are of great significance for reducing the use of chemical fungicides to control soil-borne fungal diseases, thereby improving the ecological environment, and for providing technical support for food safety and sustainable development.

## 2. Materials and Methods

### 2.1. Tested Strains

Information on the 12 bacterial and fungal strains used is listed in Table 1. *Bacillus velezensis* BV01 was isolated from a contaminated potato dextrose agar (PDA) plate in the laboratory. *Bacillus velezensis* JDF and *B*. *subtilis* L01 and BS208 were isolated from three commercially available bacterial agents NongBaoShengWu^®^, LvLong^®^, and GuanLan^®^, respectively, and the eight fungal plant pathogen strains were provided by colleagues from Beijing Academy Agriculture and Forestry Sciences, China Academy Agricultural Sciences, Guangxi Academy Agricultural Sciences, Nanjing Agricultural University, and our institute (Table 1). All strains were deposited in the China General Microbiological Culture Collection Center (CGMCC) and the State Key Laboratory of Mycology, Institute of Microbiology, Chinese Academy of Sciences.

### 2.2. Evaluation of Antagonistic Abilities In Vitro

All bacterial strains were tested for their antagonistic abilities against eight phytopathogens in dual-culture assays [18]. The bacterial strains were grown in nutrient broth (NB: peptone 10.0 g, beef paste 3.0 g, NaCl 5.0 g, dH_2_O 1000 mL) at 28 °C for 2 d, and the cell suspension was adjusted to 5 × 10^8^ CFU/mL. The fungal strains were grown on PDA at 25 °C for 5 d. Mycelial plugs (5 mm in diam.) were placed in the center of each new PDA plate (90 mm in diam.); then, four sterile filter papers (5 mm in diam.), which contained 6 μL of a bacterial suspension, were placed evenly around individual mycelial plugs. Sterile water was used as the control. All plates were incubated at 25 °C for 5 d. Each treatment had three replicates. Measurements were calculated with the following formula: y = (A − B)/A × 100% (y: percentage of inhibition, A: colony diameter of pathogen on control plate, and B: colony diameter of pathogen in experimental group) [22].

### 2.3. Assessment of Antifungal Activity of Bacterial Sterile Filtrate

The antifungal activity of bacterial sterile filtrate was evaluated by measuring the diameter of the fungal colony [23]. Four bacterial strains were incubated in NB with shaking at 180 rpm at 28 °C for 2 d, and then the cultures were centrifuged at 8000 rpm at 4 °C for 20 min to collect the supernatant. The supernatant was filtered through a 0.22 μm filter and then mixed into molten PDA at 45–50 °C with a concentration of 20% (*v*/*v*). The mycelium plug (5 mm in diam.) of each pathogen was placed at the center of each PDA plate with bacterial filtrate and incubated for 5 d at 25 °C. The PDA plate without bacterial filtrate was used as the control. Three replicates were set up for each treatment. The inhibition percentage (y%) was calculated with the following formula: y = (A − C)/A × 100% (A: growth radius of pathogen in control and C: growth radius of pathogen in different treatments).

### 2.4. Morphological Observation and Molecular Identification of Strain BV01

The morphological characteristics of strain BV01 were recorded after incubation on a nutrient agar (NA: peptone 10.0 g, beef paste 3.0 g, NaCl 5.0 g, agar 18.0 g, dH_2_O 1000 mL) plate at 28 °C for 2 d. A single colony was then taken and evenly spread onto a glass slide, dry fixed, and Gram stained for 1 min [24]. The microscopic photographs were taken with a Zeiss AxioCam MRc 5 digital camera (Carl Zeiss, Jena, Germany) attached to Zeiss Axioskop 2 plus microscope (Carl Zeiss, Göttingen, Germany).

Genomic DNA was extracted from fresh cultures using a bacterial genomic DNA kit (ZOMAN, Beijing, China) according to the manufacturer’s instructions. The *16S* rRNA and the gyrase subunit A protein (*gyrA*) regions were amplified using the primer pairs of 27F (5′-AGAGTTTGATCCTGGCTCAG-3′) and 1492R (5′-GGTTACCTTGTTACGACTT-3′) and *gyrA*-F (5′-CAGTCAGGAAATGCGTACGTCTT-3′) and *gyrA*-R (5′-CAAGGTAATGCTCCAGGCATTGCT-3′), respectively. PCR reactions were conducted using an ABI 2720 Thermal Cycler (Applied Biosciences, Foster City, CA, USA), and the products were then sequenced using an ABI3730XL DNA Sequencer (Applied Biosciences, Foster City, CA, USA). The newly obtained sequences and those of the ex-type strain as well as the related ones retrieved from GenBank were aligned using BioEdit 7.2 [25] and analyzed with the neighbor-joining (NJ) method [26] via MEGA X [27]. The topological confidence of the resulting tree and the statistical supports of the branches were tested using the neighbor-joining bootstrap proportion (NJBP) with 1000 replications, each with 10 replicates of random addition of taxa [28].

### 2.5. Determination of Enzyme Activity and Secondary Metabolites

Protease, cellulase, and β-1,3-glucanase were detected on skim milk, carboxymethyl cellulose (CMC), and glucose agar, respectively [29]. Siderophore and indole-3-acetic acid (IAA) were tested using modified chrome azurol S and Salkowski reagent agar, respectively [30]. Enzyme activity and production of metabolites were observed based on the presence of clear zones around bacterial colonies after incubation at 28 °C for 3 d. All treatments were repeated three times.

### 2.6. Biocontrol Activity of Strain BV01 In Vivo

After evaluation of the antagonistic abilities of the four bacterial strains, two strains, namely, JDF and BV01, were further tested for their biocontrol and plant-growth-promotion abilities in a pot experiment. Four germinated wheat seeds were sowed per pot containing a mixture of podsolic soil and vermiculite (*v*:*v* = 1:1). On the 14th day, the roots of the plants were punctured and inoculated nearby the wounds with pathogen mycelium blocks (5 mm in diam.). After 24 h, the roots were irrigated with 30 mL (2.5 × 10^8^ CFU/mL) of bacterial suspensions per wheat plant, and the same amount of NB was used as the control. The plants were kept in a greenhouse with a 14 h/10 h photoperiod/dark period at 26 ± 1 °C. The incidence of disease of wheat treated with BV01, JDF, and the non-treated control was recorded and calculated at 20 d after *B*. *sorokiniana* inoculation. Infection types (ITs) of *B*. *sorokiniana* on wheat were evaluated based on the area of a brown or black lesion at the stem base, with scores varying from 0 to 4 (IT0: no lesion, IT1: coverage of necrotic lesion less than 1/4, IT2: lesion coverage between 1/4 and 1/2, IT3: coverage between 1/2 and 2/3, and IT4: coverage between 2/3 and total). The disease index = ∑ (d_i_ × l_i_) / (L × di_max_) × 100% (d_i_: infection type, li: number of plants with each infection type, and L: number of wheat plants investigated) [31].

Three true leaves at the age of 40 d were detached from pepper seedlings and punctured in two places with a sterile needle on both sides of the leaf. Mycelium blocks (5 mm in diam.) of *F*. *graminearum* were placed on the two wounds and then sprayed with 2 mL of either BV01 or JDF fermentation broth at a concentration of 2.5 × 10^8^ CFU/mL, and the same volume of NB was used as the non-inoculated control at 24 h after *F*. *graminearum* inoculation. The treatments were kept in a greenhouse at 26 ± 1 °C with a 14 h/10 h photoperiod/dark period for 6 d; sterile water was added once to keep the treatment moist. There were 3 leaves in each pot and 3 replicates in each treatment. The diameters of diseased spots were recorded and calculated at the 6th day after *F*. *graminearum* inoculation. The inhibition rate was calculated as follows: (D_CK_ − D_i_)/D_CK_ × 100% (D_CK_: the control group’s average colony diameter and Di: the treatment group’s average colony diameter).

### 2.7. Plant-Growth-Promoting Assays in a Greenhouse

Sterilized wheat and pepper seeds were soaked in suspensions of BV01 or JDF at a concentration of 2.5 × 10^8^ CFU/mL and then in sterile water for 10 min. NB was used as the control. The plants were kept in a greenhouse with a 14 h/10 h photoperiod/dark period at 26 ± 1 °C. The wheat and peppers were harvested at the 21st and 35th days, respectively. Plant height, fresh weight, dry weight, and leaf width were recorded, and the strong seedling index (SSI) was calculated [32].

### 2.8. Statistical Analysis

Statistical analysis was performed using SPSS 21 (Armonk, NY, USA). ANOVA was performed, and mean values were compared using Duncan’s multiple range test with *p* < 0.05 as the level of significance. All analyses were conducted using GraphPad Prism 8 (San Diego, CA, USA).

## 3. Results

### 3.1. Inhibitory Effects of Four Tested Bacterial Strains against Eight Fungal Phytopathogens

**Figure 1:**
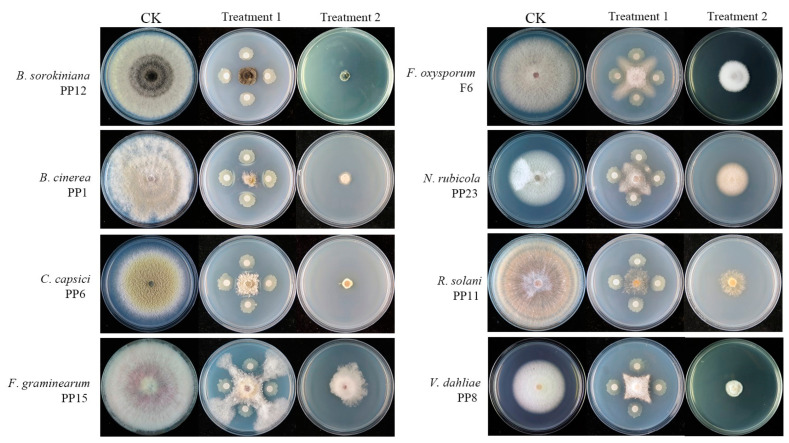
Inhibitory effects of BV01 against fungal phytopathogens. CK: only pathogen on PDA at 25 °C for 5 d; Treatment 1: dual culture of BV01 against pathogen on PDA at 25 °C for 5 d; Treatment 2: pathogen on PDA amended with fermentation broth of BV01 at 25 °C for 5 d.

Strain BV01 exhibited varying degrees of antagonism against different phytopathogens, and the inhibition rates ranged from 57% to 83% (Table 2) with the highest potential inhibitory effects against *B*. *cinerea* PP1 (Figure 1, Treatment 1). The inhibition rates showed that BV01 had significantly higher inhibitory effects than JDF, L01, and BS208 on six of the eight tested fungal phytopathogens.

Antifungal assay by fermentation broth test showed that BV01 had relatively high inhibitory effects against different pathogens (Table 2), and the highest inhibitory rate reached 92% (against *B*. *sorokiniana* PP12) (Figure 1, Treatment 2). Overall, the effects of BV01 were better than those of JDF and L01 and were significantly superior to those of BS208.

### 3.2. Identification of Strain BV01

**Figure 2:**
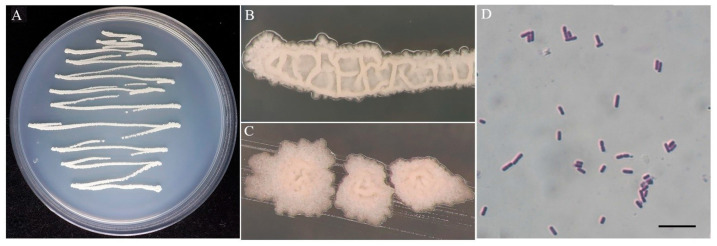
Colony and microscopic characteristics of BV01. (**A**–**C**) General colonies on nutrient agar; (**D**) Gram-stained cells. Bar: D = 10 µm.

**Figure 3:**
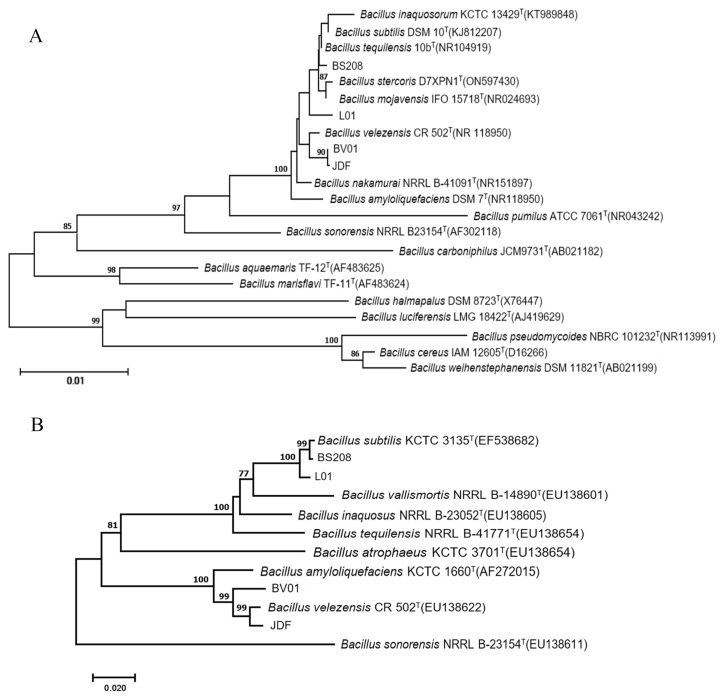
Phylogenetic trees generated based on sequences of *16S* rRNA (**A**) and *gyrA* (**B**) regions of *Bacillus* species. NJBP values greater than 75% are shown at the nodes.

The colony of BV01 was ivory white and non-transparent with a rough surface on NA medium (Figure 2A–C). The cells were Gram-positive (Figure 2D), rod-shaped, 1.43–2.53 µm long, 0.66–0.88 µm wide, and occurred singly, in pairs, or occasionally in short chains. The analysis of *16S* rRNA sequences showed that strain BV01 shared 99% identity with the type strain of *B. velezensis* (CR502) according to a BLAST search. The resulting NJ trees based on sequences of *16S* rRNA and *gyrA* (Figure 3) showed that BV01 clustered with *Bacillus* species and grouped with the type strain of *B. velezensis*, which confirmed its taxonomic position.

### 3.3. Detection of Antagonism-Related Lytic Enzymes

**Figure 4:**
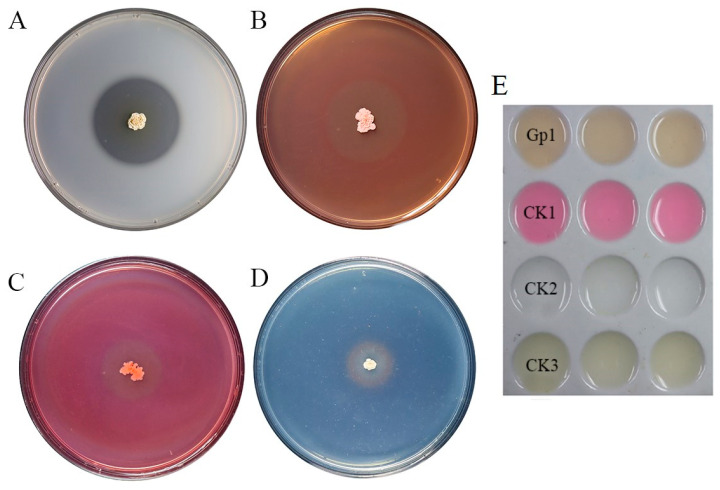
Detection of extracellular enzyme production and growth-promotion traits of BV01. (**A**) protease; (**B**) cellulase; (**C**) β-1,3-glucanase; (**D**) siderophore; (**E**) indole-3-acetic acid (IAA); Gp_1_: BV01 suspension, CK_1_: 10 mg/mL IAA, CK_2_: sterilize distilled water, CK_3_: NB.

Clear zones detected around the colony of BV01 indicated that the strain produced protease, cellulase, and β-1,3-glucanase (Figure 4A–C) as well as siderophore (Figure 4D) and IAA (Figure 4E), which suggested its high potential in biological control. The production of IAA reached 12.17 mg/mL after incubation for 6 d.

### 3.4. Biocontrol Effects of Bacterial Strains BV01 and JDF on Wheat Root Rot

**Figure 5:**
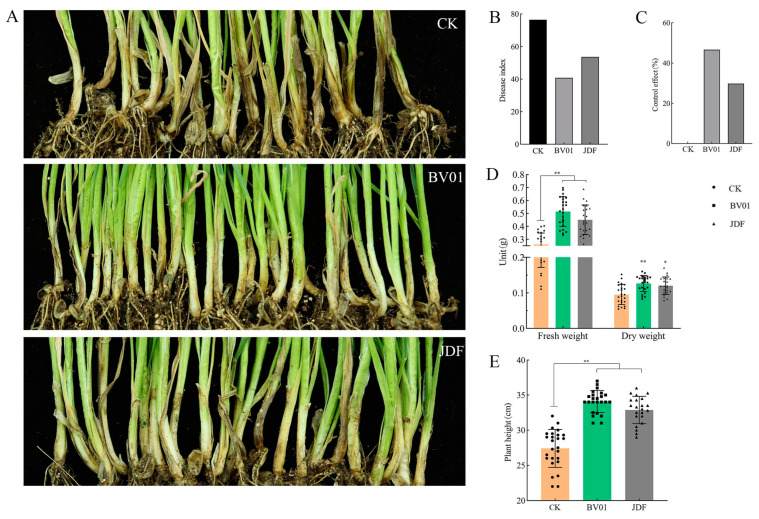
Inhibitory effect of *Bacillus* strains on wheat root rot disease caused by *B. sorokiniana* PP12. (**A**) Symptoms of *B. sorokiniana* on wheat roots with different treatments; (**B**) disease index of *B. sorokiniana* in the treatments with BV01 and JDF; (**C**) inhibition rates of BV01 and JDF; (**D**) fresh and dry weights of wheat; (**E**) shoot biomass (cm) measured by plant height of wheat. The results were observed after 40 d of incubation. Values are the means ± SEs, *n* = 27 plants, ** *p* < 0.001, and * *p* < 0.05.

Lesions at the stem bases of wheat were obviously brown in the non-treated control, while those treated with BV01 and JDF were very slightly infected (Figure 5A). The disease indices of CK, BV01, and JDF were 76.4, 40.8, and 53.6, respectively (Figure 5B). In the BV01 treatment, infection with wheat root rot was significantly (*p* < 0.05) reduced, the relative control efficacy was 47% (Figure 5C), and the fresh and dry weights (Figure 5D) and plant height (Figure 5E) were increased by 91%, 34%, and 24%, respectively.

### 3.5. Biocontrol Effect of Strain BV01 on Fusarium Wilt

**Figure 6:**
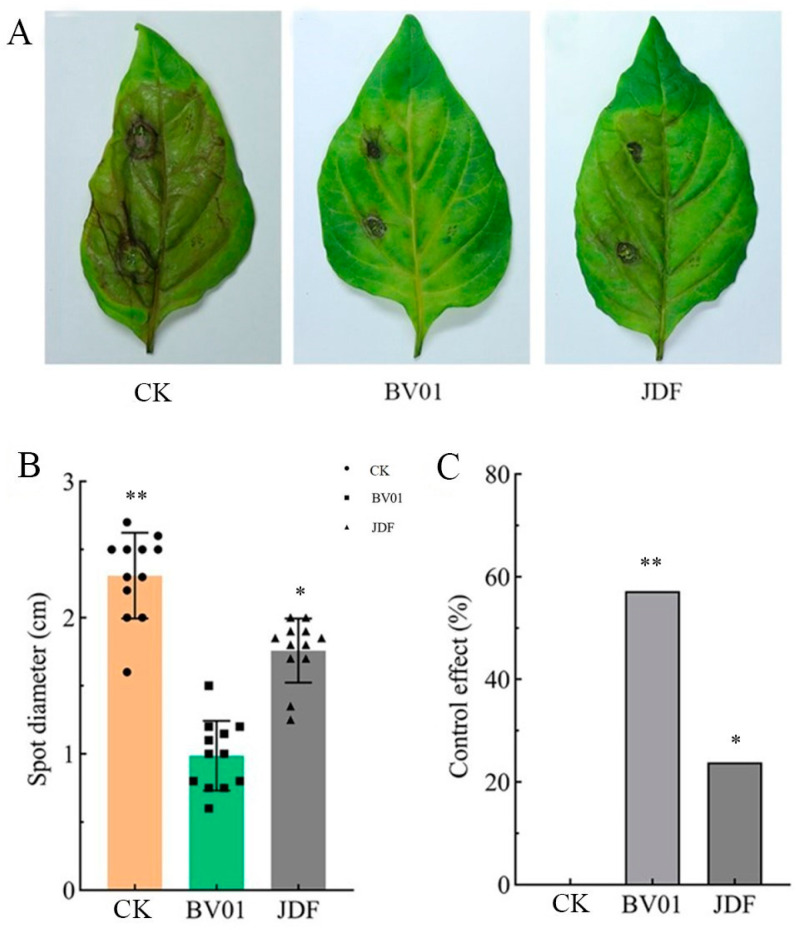
Effect of *Bacillus* strains on disease symptoms caused by *F*. *graminearum* PP15 on leaves. (**A**) Symptoms of *F*. *graminearum* on leaves with different treatments; (**B**) spot diameter after treatment with BV01 or JDF; (**C**) inhibition rates of BV01 and JDF. Values are the means ± SEs, *n* = 9 leaves, ** *p* < 0.001, and * *p* < 0.05.

The symptoms on pepper leaves of the control were severe, on those treated with JDF were moderate, and on those treated with BV01 were weak (Figure 6A). The average diameter of a spot was 2.31, 0.99, and 1.76 cm in the CK, BV01, and JDF treatments (Figure 6B). The control effect reached 57% and 24% for the treatments with BV01 and JDF, respectively (Figure 6C).

### 3.6. Growth-Promotion Effects of Strain BV01 on Wheat and Pepper

**Figure 7:**
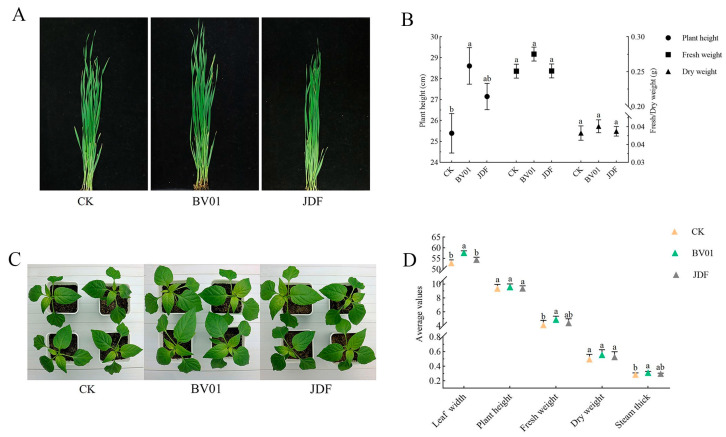
Growth-promotion effects of BV01 and JDF on wheat and pepper. Wheat growth (**A**) and experimental conditions (**B**) in CK, BV01, and JDF pot experiments, *n* = 12 plants. Pepper growth (**C**) and experimental conditions (**D**) in CK, BV01, and JDF pot experiments, *n* = 9 plants. Values are the means ± SEs, different letters show significant differences (Fisher’s LSD, *p* < 0.05).

Wheat treated with BV01 exhibited an increase in height of 13% (Figure 7A), while the fresh weight and dry weight were improved by 10% and 5% (Figure 7B). Pepper treated with BV01 exhibited increases in the fresh weight, leaf width, and stem thickness of 20%, 9%, and 9%, respectively. The dry weight and plant height were improved by 12% and 2% (Figure 7C,D). The SSI for treatment with BV01 and JDF increased by 19% and 10%. These findings suggest that strain BV01 was more effective in promoting plant growth than JDF.

## 4. Discussion

For a long time, *Bacillus amyloliquefaciens* and *B*. *subtilis* were known to have biocontrol functions against various plant pathogens [33]. Recently, *B. velezensis* was reported as a biocontrol agent against many phytopathogens. For example, *B*. *velezensis* strain F21 can control *Fusarium* wilt on watermelon [19], and strain BR-01 has strong antagonistic effects on rice pathogens [34], while strain CE100 increases fruit yield of strawberries by controlling fungal diseases [35]. The star strain FZB42 was initially established in 1998, and successive studies on its antimicrobial substances, interactions between plants and bacteria, regulatory small RNAs, and biocontrol enzymes have been carried out [33]. In previous studies, antagonistic strains of *B*. *velezensis* were often isolated from water, soil, air, plant roots, plant surfaces, and animal intestines [7]. In the present study, strain BV01 was derived from a PDA plate in the laboratory and speculated to be an air source strain. Based on morphological characteristics and phylogenetic evidence, strain BV01 was identified as *B*. *velezensis*; further exploration of its biological control potential was then performed. Its dual-culture inhibition rates against different pathogens were greater than 56%, and the fermentation broth inhibition rates were reduced by more than 36% when compared to the control. The results indicate that BV01 produces a special antibacterial substance. Some lipopeptide extract components of *B*. *amyloliquefaciens* have been demonstrated as key substances in controlling the growth of *Xanthomonas citri* subsp. *citri* [36]. Zhou et al. [34] proved that the relative inhibition rate of *B*. *velezensis* BR-01 against *F*. *fujikuroi* was 57%, while the strain showed no antagonistic ability against *R*. *solani.* The results of the current study revealed that strain BV01 possessed very strong antagonistic activity and broad-spectrum biological ability against *B*. *cinerea*, *F*. *oxysporum*, *C*. *capsici*, *V*. *dahliae*, *R*. *solani*, *B*. *sorokiniana*, *F*. *graminearum*, and *N*. *rubicola*.

Many *Bacillus* species produce a variety of hydrolytic enzymes, such as cellulase, β-1,3-glucanase, and protease, which are responsible for the degradation of diverse components of fungal pathogens [35,37]. The detection of cellulase, protease, and β-1,3-glucanase in BV01 supports its association with the growth suppression of several fungal phytopathogens. Our results also revealed that strain BV01 effective in vitro against fungal pathogens was also able to produce siderophores, which are related to indirect antagonistic processes such as plant defenses and growth promotion [30]. Moreover, some members of *Bacillus* invade the rhizosphere of plants and promote plant growth by producing plant hormones, such as IAA, cytokinins, and gibberellins, and chelating minerals and siderophores. Many plant-growth-promoting bacteria produce IAA, which promotes the development of plant roots, and are usually utilized as bioinoculants [38,39,40,41,42,43,44,45]. In a previous study, *B*. *velezensis* BY6 was reported to significantly increase the dry and fresh mass and plant height of Pdpap poplar seedlings [46]. In the present study, *B. velezensis* BV01 produced IAA during its growth. Moreover, our pot experiment results revealed that pepper and wheat treated with strain BV01 possessed higher fresh weight, dry weight, plant height, leaf width, stem thickness, and SSI than controls. Both the antifungal activity assay and greenhouse pot experiment indicated that the strain BV01 has biocontrol and plant-growth-promotion potential.

Wheat and pepper are two of the most commonly grown crops and vegetables in the world. Several pathogens cause severe diseases of them and thus reduce significantly their yields. For example, wheat root rot caused by *B*. *sorokiniana*, *Fusarium* spp., and other pathogens alone or in combination generally can lead to wheat yield reductions of 20%–30%, with severe cases of more than 50% [47,48]. Previous studies revealed that *B*. *subtilis* and *B. amyloliquefaciens* can prevent and control wheat root rot [47]. However, there are few studies on the effects of *B*. *velezensis* on wheat root rot caused by *B*. *sorokiniana*. *Bacillus velezensis* strains CC09 and NEAU-242-2 could be used as potential biocontrol agents to control wheat disease [49,50]. In this study, *B. velezensis* strain BV01 was able to effectively control wheat root rot caused by *B*. *sorokiniana* in a greenhouse, with a control rate of 47%. The occurrence of pepper wilt is increasing currently and seriously affects the quality of pepper. For example, the incidence of pepper wilt disease in China is generally 15%–30%, with severe cases decreasing quality by 70%–80% [51]. The main pathogen, *Fusarium graminearum*, is a highly destructive phytopathogen, not only lowering crop yields but also producing mycotoxins and affecting crop quality. Previous studies have confirmed that *B*. *velezensis* could control pepper root rot [52], wheat spikes [53], corn stalk rot [54], and corn head blight [55]. To our knowledge, the present study is the first report that *B*. *velezensis* can serve as a potential biocontrol agent for controlling pepper wilt induced by *F*. *graminearum*. *Bacillus velezensis* BV01 not only promotes the growth of wheat and pepper seedlings but also significantly controls wheat root rot and pepper wilt. In summary, *Bacillus velezensis* BV01 has good control effects in both dual-culture and fermentation broth tests against *B*. *sorokiniana* and *F*. *graminearum*, and it obviously reduced the disease symptoms and promoted the growth of wheat and pepper.

## 5. Conclusions

*Bacillus velezensis* BV01 showed protease, cellulase, and β-1,3-glucanase activities, which are related to phytopathogen cell wall degradation, and produced growth-promotion substances such as IAA and siderophore. This strain also suppressed the growth of eight phytopathogens both in dual-culture and sterile filtrate assays and significantly reduced the disease incidence of wheat root rot and *Fusarium* wilt in greenhouse settings. Moreover, it significantly promoted wheat and pepper growth. In conclusion, BV01 exhibits broad and effective antagonistic activity against several phytopathogens, promotes plant growth, and is worthy of further exploration of its biocontrol applications in eco-friendly agriculture practices.

## Figures and Tables

**Table 1:** The bacterial and fungal strains tested in this study.

Strain	Characteristics Relevant to This Work	Source
*Bacillus velezensis* CGMCC 1.60184	Isolated from the State Key Laboratory of Mycology, Institute of Microbiology, Chinese Academy of Sciences (116.38982° E, 40.01076° N) on 6 November 2019	This work
*B. velezensis* JDF	Registration of broad-spectrum antagonism, stress resistance and growth-promoting ability, used as a positive control	Isolated from NongBaoShengWu^®^ bacterial agent
*B. subtilis* L01	Registration of antimicrobial activity and strong stress resistance, used as a positive control	Isolated from LvLong^®^ bacterial agent
*B. subtilis* BS208	Registration of prevention and control of gray mold and powdery mildew, used as a positive control	Isolated from GuanLan^®^ bacterial agent
*Bipolaris sorokiniana* PP12	Causes wheat root rot	Provided by Prof. Niu Yongchun of Chinese Academy of Agricultural Sciences
*Botrytis cinerea* PP1	Causes tomato gray mold	Provided by Prof. Qiu Jiyan of Beijing Academy of Agricultural Sciences
*Colletotrichum capsici* PP6	Causes ring rot disease	
*Fusarium graminearum* PP15	Causes *Fusarium* head blight	Provided by Prof. Ma Zhengqiang of Nanjing Agricultural University
*F. oxysporum* F6	Causes blight disease	Provided by Prof. Li Qili of Guangxi Academy of Agricultural Sciences
*Neocosmospora rubicola* PP23	Causes root and stem rot	Provided by Dr. Fu Shenzhan of Institute of Microbiology, Chinese Academy of Sciences
*Rhizoctonia solani* PP11	Causes wilt disease	Provided by Prof. Niu Yongchun of Chinese Academy of Agricultural Sciences
*Verticillium dahliae* PP8	Causes greensickness	

**Table 2:** Antifungal activities of four bacterial strains.

Strains	Dual-Culture Inhibition Rate (%)				Fermentation Broth Inhibition Rate (%)			
	CGMCC 1.60184	JDF	L01	BS208	CGMCC 1.60184	JDF	L01	BS208
*B. sorokiniana* PP12	80.68 ± 1.23 ^a^	79.55 ± 0.93 ^a^	77.27 ± 1.05 ^b^	27.27 ± 2.47 ^c^	92.26 ± 0.68 ^a^	76.77 ± 0.23 ^b^	70.32 ± 1.03 ^c^	8.39 ± 1.89 ^d^
*B. cinerea* PP1	82.95 ± 1.23 ^b^	85.23 ± 2.23 ^a^	85.23 ± 2.14 ^a^	0.00 ± 0.83 ^c^	81.82 ± 0.82 ^a^	64.77 ± 1.00 ^c^	71.59 ± 0.58 ^b^	2.27 ± 1.44 ^d^
*C. capsici* PP6	79.55 ± 0.64 ^a^	75.00 ± 1.25 ^b^	77.27 ± 1.07 ^b^	26.14 ± 1.68 ^c^	72.73 ± 0.75 ^a^	71.59 ± 0.86 ^a^	72.73 ± 0.42 ^a^	4.55 ± 1.45 ^b^
*F. graminearum* PP15	61.36 ± 1.09 ^b^	65.91 ± 0.49 ^a^	62.50 ± 0.62 ^b^	0.00 ± 0.62 ^c^	59.09 ± 1.66 ^a^	43.18 ± 0.52 ^c^	47.53 ± 0.66 ^b^	13.64 ± 0.97 ^d^
*F. oxysporum* F6	65.91 ± 0.66 ^a^	62.50 ± 1.03 ^b^	52.27 ± 0.71 ^c^	32.95 ± 1.27 ^d^	56.79 ± 1.23 ^a^	13.58 ± 1.06 ^b^	12.04 ± 0.69 ^b^	8.95 ± 0.46 ^c^
*N. rubicola* PP23	56.52 ± 1.02 ^a^	50.72 ± 0.93 ^b^	50.72 ± 1.23 ^b^	42.03 ± 1.24 ^c^	36.13 ± 1.19 ^a^	12.18 ± 0.99 ^b^	12.18 ± 0.52 ^b^	4.19 ± 0.74 ^c^
*R. solani* PP11	69.32 ± 0.71 ^a^	54.55 ± 0.86 ^b^	53.41 ± 1.23 ^b^	0.00 ± 1.7 ^c^	46.59 ± 1.23 ^a^	0.00 ± 1.08 ^c^	0.00 ± 1.21 ^c^	5.68 ± 0.56 ^b^
*V. dahliae* PP8	70.15 ± 1.18 ^a^	58.21 ± 0.73 ^b^	55.22 ± 1.35 ^c^	32.84 ± 0.88 ^d^	71.72 ± 1.18 ^a^	59.07 ± 0.73 ^b^	61.03 ± 1.35 ^b^	33.95 ± 0.88 ^c^

The inhibition rates (%) (*n* = 3, mean ± SE). Different letters indicate significantly different groups (*p* < 0.05, ANOVA, Tukey HSD).

## Data Availability

All the data relevant to this manuscript are available on request from the corresponding author.
